# Measuring Action Potential Propagation Velocity in Murine Cortical Axons

**DOI:** 10.21769/BioProtoc.4876

**Published:** 2023-11-05

**Authors:** Oron Kotler, Yana Khrapunsky, Ilya Fleidervish

**Affiliations:** Dept. of Physiology and Cell Biology, Faculty of Health Sciences and Zelman Center for Neuroscience, Ben–Gurion University of the Negev, Beer Sheva 84105, Israel

**Keywords:** Action potential, Action current, Pyramidal neuron, Neocortex, Propagation velocity, Trigger zone, Backpropagation, Loose patch, Whole-cell recording

## Abstract

Measuring the action potential (AP) propagation velocity in axons is critical for understanding neuronal computation. This protocol describes the measurement of propagation velocity using a combination of somatic whole cell and axonal loose patch recordings in brain slice preparations. The axons of neurons filled with fluorescent dye via somatic whole-cell pipette can be targeted under direct optical control using the fluorophore-filled pipette. The propagation delays between the soma and 5–7 axonal locations can be obtained by analyzing the ensemble averages of 500–600 sweeps of somatic APs aligned at times of maximal rate-of-rise (dV/dtmax) and axonal action currents from these locations. By plotting the propagation delays against the distance, the location of the AP initiation zone becomes evident as the site exhibiting the greatest delay relative to the soma. Performing linear fitting of the delays obtained from sites both proximal and distal from the trigger zone allows the determination of the velocities of AP backward and forward propagation, respectively.

Key features

• Ultra-thin axons in cortical slices are targeted under direct optical control using the SBFI-filled pipette.

• Dual somatic whole cell and axonal loose patch recordings from 5–7 axonal locations.

• Ensemble averaging of 500–600 sweeps of somatic APs and axonal action currents.

• Plotting the propagation delays against the distance enables the determination of the trigger zone's position and velocities of AP backward and forward propagation.

## Background

Neurons generate action potentials (APs), all-or-none electrical events propagating along their processes by eliciting transient changes in membrane permeability (Hodgkin and Huxley, 1952). One of the most critical AP parameters, propagation velocity, was first measured in peripheral axons in the second half of the 19th century (Helmholtz, 1850; Bernstein, 1868). Measuring the propagation velocity in ultra-thin central axons became possible only recently, as it requires previously unachievable temporal and spatial resolution. Several studies have attempted to measure propagation delays using simultaneous whole-cell recordings from soma and giant axonal blebs (Shu et al., 2007; Kole et al., 2007) or dual whole-cell, cell-attached recordings from the soma and axonal trunk (Sasaki et al., 2012). Optical recordings from neurons stained with voltage-sensitive dyes were used to determine the AP initiation site and describe the AP propagation pattern and velocity (Palmer and Stuart, 2006; Popovic et al., 2011). We describe how to measure AP propagation velocity in fluorescently labeled cortical axons in acute brain slice preparation by recording whole-cell somatic voltage and action currents from multiple axonal locations using a loose patch configuration. We previously employed this method in cortical brain slices (Baranauskas et al., 2013; Kotler et al., 2023). We believe that, with minor adjustments, this method can be applied to determine the AP propagation speed in various brain regions and experimental setups.

Accurate measurement of both distance and time delays is crucial to calculate propagation velocity. Measuring the distance poses fewer technical challenges once the location of the AP trigger zone is known. Thus, not establishing the trigger zone position before attempting to determine the propagation velocity could lead to inaccurate conclusions, given that the propagation delay between the soma and the axonal segment located 100 µm away is almost zero. Accurately measuring the time delays presents a greater challenge since it demands sufficient temporal and amplitude resolution in action current recording. For adequate temporal resolution, we suggest widening the bandwidth of the axonal voltage clamp recording to 20 kHz. Spike-triggered averaging of 500–600 sweeps should be sufficient to resolve action currents with a signal-to-noise ratio above 3.

## Materials and reagents

**Biological materials**

Experimental animals: ICR mice, P6 or older (Envigo, Israel), are suitable for brain slice experiments. Any other mouse strain could be used. All animal experiments should be performed in accordance with institutional and national guidelines for the care and use of laboratory animals.

**Materials**

1. Whatman qualitative filter paper, Grade 1, circles, diam. 90 mm (Sigma-Aldrich, catalog number: WHA1001090)
2. Petri dishes, polystyrene, size 100 mm × 15 mm (Sigma-Aldrich, catalog number: P5856)
3. Feather double-edge carbon steel blades (Ted Pella, catalog number: 121-9)
4. GEM single edge, carbon steel blades, uncoated (Ted Pella, catalog number: 121-1)
5. PELCO Pro CA44 instant tissue adhesive (Ted Pella, catalog number: 10033)

**Reagents**

1. Sodium-binding benzofuran isophthalate (SBFI, Tetraammonium Salt, cell impermeant) (Invitrogen, catalog number: S1262)
2. Sodium chloride (NaCl) (Biolab, CAS number: 7647-14-5)
3. Potassium chloride (KCl) (Sigma-Aldrich, CAS number: 7447-40-7)
4. Calcium chloride dihydrate (CaCl_2_) (Sigma-Aldrich, CAS number: 10035-04-8)
5. Magnesium sulfate heptahydrate (MgSO_4_) (Sigma-Aldrich, CAS number: 10034-99-8)
6. Sodium phosphate monobasic monohydrate (NaH_2_PO_4_) (Acros Organics, CAS number: 10049-21-51)
7. Sodium bicarbonate (NaHCO_3_) (Biolab, CAS number: 144-55-8)
8. D-glucose (Sigma-Aldrich, CAS number: 50-99-7)
9. Potassium D-gluconate (Sigma-Aldrich, CAS number: 299-27-4)
10. Magnesium chloride anhydrous (MgCl_2_) (Sigma-Aldrich, CAS number: 7786-30-3)
11. HEPES (Sigma-Aldrich, CAS number: 7365-45-9)
12. Distilled water (H_2_O)
13. Isoflurane (Piramal Critical Care, Bethlehem, PA, USA)

**Solutions**

1. Artificial cerebrospinal fluid ×10 stock solution (aCSFx10) (see Recipes)
2. Artificial cerebrospinal fluid solution (aCSF)-working solution (see Recipes)
3. MgSO_4_ 1 M stock (see Recipes)
4. CaCl_2_ 1 M stock (see Recipes)
5. K^+^-based intracellular solution (see Recipes)

**Recipes**

1. **Artificial cerebrospinal fluid ×10 stock solution (aCSFx10)**
  **Table:** 

Reagent	Final concentration (mM)	m.w.	Amount (g) for 1 L
NaCl	1,240	58.44	72.466
KCl	30	74.56	2.237
NaHCO_3_	260	84.01	21.843
NaH_2_PO_4_	12.5	137.99	1.725
2. **Artificial cerebrospinal fluid solution (aCSF)-working solution**
  **Table:** 

Reagent	Final concentration	Amount for 1 L
aCSFx10	n/a	100 mL
D-glucose 1 M	10 mM	10 mL
MgSO_4_ 1 M	2 mM	2 mL
H_2_O	n/a	~850 mL
Bubble with 95% O_2_-5% CO_2_ gas mixture for 15 min		
CaCl_2_ 1 M	2 mM	2 mL
Add H_2_O up to 1 L exactly
  Remark: Bubbling with 95% O_2_-5% CO_2_ gas mixture is necessary to avoid precipitation of the divalent ions.
3. **MgSO_4_ 1 M stock**
  **Table:** 

Reagent	Final concentration	Amount, g for 50 mL
MgSO_4_	1 M	10.165
Add H_2_O up to 50 mL exactly
4. **CaCl_2_ 1 M stock**
  **Table:** 

Reagent	Final concentration	Amount (g) for 50 mL
CaCl_2_	1 M	7.352
Add H_2_O up to 50 mL exactly
5. **K^+^-based intracellular solution**
  **Table:** 

Reagent	Final concentration	Amount for 100 mL
Potassium D-gluconate (m.w. 234.25)	130	3.045 g
KCl 1 M	6	0.6 mL
MgCl_2_ 1 M	2	0.2 mL
HEPES 1 M	10	1 mL
NaCl 1 M	4	0.4 mL
H_2_O	n/a	to 100 mL
Titrate to pH 7.25 with KOH 1 M
  Due to the large size and spatial complexity of L5 pyramidal neurons in slices, ATP and GTP supplements were not added to the intracellular solution. The freshly prepared K^+^-based intracellular solution was tested in whole-cell recording from several neurons before supplementing with SBFI. The SBFI-containing solution was separated in aliquots of 100 µL into small Eppendorf microtubes and stored at -20 °C.

## Equipment

1. Upright microscope equipped with IR-DIC optics (Olympus, model: BX51WI)
2. Water-immersion objective lens (magnification: 60×, numerical aperture: 1.0, working distance: 2 mm) (Olympus, LUMPLFLN60XW, product number: N2667800)
3. Shifting table (Luigs & Neumann, model: V380FM), control box (Luigs & Neumann, model: SM7)
4. Two LN mini manipulators (Luigs & Neumann, model: Mini 25)
5. Slice mini chamber with temperature controller (Luigs & Neumann, 200-100 500 0150-S and 200-100 500 0145)
6. U-shaped platinum grid to weigh down a slice in a recording chamber
7. C2400 CCD Camera (Hamamatsu)
8. NeuroCCD-SMQ camera, 0.38× coupler (to achieve the final magnification of 1 pixel = 1 µm with 60× objective), and NeuroPlex software (Redshirt Imaging)
9. High Power Collimated LED Light Source, 385 nm (Prizmatix, model: Mic-LED-385)
10. Modified filter set, dichroic mirror 400 nm, long pass emission filter 420 nm (Olympus, model: U-MNU2)
11. MultiClamp-700B amplifier, equipped with two CV-7B headstages (Molecular Devices)
12. Semiautomatic microtome with a vibrating blade (Leica Biosystems, model: VT-1200) with Vibrocheck unit for adjustment of vertical deflection of the blade
13. Analytical balance (MRC-Laboratory Equipment, model: ASB-220-C2)
14. Bench pH meter (Hanna Instruments, model: HI2211)
15. Small scissors, tweezers, spatula, Pasteur pipette
16. Micropipette puller (Sutter Instrument, model: P-97)
17. Ultima IV 2P microscope (Bruker) equipped with Mai Tai DeepSee laser (Spectra Physics)
18. Semi-Automatic Vibrating Blade Microtome (Leica Biosystems, model: VT-1200)

## Software and datasets

1. pCLAMP 9 Electrophysiology Data Acquisition & Analysis Software (Molecular Devices, Version 9.2.1.9, 2007) for data acquisition; pCLAMP 11 Electrophysiology Data Acquisition & Analysis Software (Molecular Devices, Version 11.2.2.17, 2022) for data analysis
2. IDL (Exelis Visual Information Solutions, Version 8.3, 2013) and NeuroPlex (RedShirt Imaging, Version 10.2.2, 2011)
3. ImageJ (Version 1.54f 29, 2023)
4. Excel (Microsoft Office 365, 2022)

## Procedure

- **Preparation of brain slices**
  Coronal or sagittal slices are suitable, as these plains of section preserve the axonal and apical dendritic tree of L5 pyramidal neurons.
  1. Preparing the solution for brain exposure and slicing:
    - Transfer 250 mL of freshly prepared aCSFx1 solution into a 300 mL chemical glass beaker.
    - Place the glass beaker into the ice box.
    - Bubble the aCSFx1 solution with 95% O_2_-5% CO_2_ gas mixture for at least 15 min to attain equilibrium in gas partial pressures.
    - Cover the glass beaker with Parafilm.
    - Cool the solution to 4 °C.
    - Add 0.7 mL of MgSO_4_ 1 M.
    - Add 0.25 mL of CaCl_2_ 1 M (prior bubbling with 95% O_2_-5% CO_2_ gas mixture is necessary to prevent Ca^2+^ ions precipitation).
    - Cover the chemical glass with Parafilm.
  2. Preparing the incubation solution for brain slices:
    - Transfer 500 mL of freshly prepared aCSFx1 solution into the slice incubator.
    - Bubble with 95% O_2_-5% CO_2_ for at least 15 min.
    - Cover the slice incubator with Parafilm.
    - Warm the aCSFx1 solution to 30 °C.
    - Add 0.7 mL of CaCl_2_ 1 M.
  3. Preparing the vibratome:
    - Put the black buffer tray and specimen holder into the freezer (-20 °C).
    - Take the double-edge feather blade, wash it with 70% ethanol, rinse with water, and wipe.
    - Insert the blade into the blade holder.
    - Insert the Vibrocheck unit into its slot and connect to the vibratome by the USB cable.
    - Wait for the appearance of a green light on the *Down* button.
    - Insert the Allen screwdriver into the blade holder slot and rotate it 90° counterclockwise until a white strip appears on the left side.
    - Push the *Down* button.
    - Push the *Run* button.
    - If the display shows a value different from 0 (e.g., -0.1), push the *Stop* button.
    - Release the blade.
    - Insert the screwdriver in the adjustment hole opening and turn in the "-" direction.
    - Push the *Run* button.
    - Repeat if the display shows a value different from 0.
    - After reaching 0, push the "Down" button to remember the parameters.
    - Move the blade to the top position and remove the Vibrocheck block.
  4. Slicing procedure (done in the chemical hood):
    - Put the Whatman filter paper 90 mm into the 100 mm Petri dish and place the dish on ice.
    - Prepare the single edge, carbon steel blade.
    - Prepare surgical instruments: scissors, small mini-scissors, tweezers, spatula, and Pasteur cut pipette.
    - Bring the mouse from the animal facility.
    - Check the temperature of the solutions.
    - Take the black buffer tray and specimen holder from the freezer and place the black buffer tray on ice.
    - Wipe the specimen holder.
*Note: The holder should be completely dry.*
    - Place a small amount of instant tissue adhesive on the center of the specimen holder.
    - Fill the Petri dish with cold slicing solution (step A1).
    - Put two weighing dishes near the surgical instruments.
    - Fill two weighing dishes with cold slicing solution.
    - The next steps have to be performed in accordance with the Institutional and National Animal Care regulations.
    - Measure 0.5 mL of isoflurane and distribute it over the towel attached to the cover of the exicator.
    - Cover the exicator.
    - Put the animal into the exicator and quickly cover it.
    - Wait a minute for the isoflurane to take effect.
    - Take a mouse and cut the skin from its head to expose the skull.
    - Cut the head close to occipital bone with scissors.
    - Wash the head in the first weighing dish with cold slicing solution for a few seconds.
    - Cut the bone with surgical scissors as follows:
      i. Insert the surgical scissors through the foramen magnum in the occipital bone.
      ii. Continue along the occipital bone until the end of the lacrimal bone.
      iii. Cut down from the lacrimal's end to the eye cavity.
    - Gently remove the cut bone with tweezers and expose the brain.
    - Put the brain into the second weighing dish with ice-cold slicing solution.
    - Transfer the brain into a Petri dish.
    - Cut the block of the brain (coronal or sagittal plane).
    - Paste it in the specimen holder.
    - Transfer the holder to a buffer tray.
    - Insert the buffer tray into the insert in the vibratome.
    - Fill the buffer tray with an ice-cold slicing solution.
    - Cut the first thick slice.
    - Discard the slice.
    - Adjust to 300 µm slice thickness and cut the slice.
    - Remove the slice with a paintbrush.
    - Collect the slice with a cut Pasteur pipette.
    - Transfer the slice to a warm incubation solution (step A2).
    - Repeat until the desired number of slices is cut.
    - Incubate the slices for 60 min.
    Caution: It is advisable to perform steps 4r–4ee with maximum speed, ideally completing them in under 1 min. More extended periods of ischemia/hypoxia could cause irreversible damage to the brain tissue.
- **Whole-cell recording and filling neurons with fluorescent dye (always document the experiment in a lab book)**
  1. Turn on all equipment and perfuse aCSFx1 through the slice mini chamber.
  2. Set the temperature under the objective to 32 °C using the temperature controller.
  3. Transfer a 300 µm thick slice from the incubating chamber to the slice mini chamber and put a platinum grid to pin down the slice.
  4. Focus on Layer 5 cell body layer (approximately 500–700 µm below the pial surface).
  5. Identify an L5 Pyramidal neuron to patch using the 60× objective. Do not move the X and Y position of the microscope from this point on.
  6. Pull two pipettes by using the P-97 micropipette puller. Note that the pipette resistance should be 10–12 MΩ when filled with K^+^-based intracellular solution or 7–9 MΩ when filled with aCSF. Note that optimal pipette size may vary depending on the specific preparations.
  7. Cut a 4–5 mm wide, 10 mm long strip of Parafilm. Wrap the pipette with one or two layers of Parafilm, ~1 mm from the tip, to reduce the pipette stray capacitance.
  8. Fill the first pipette with K^+^-based intracellular solution supplemented with SBFI (2 mM).
  9. Fill the other pipette with aCSFx1 supplemented with SBFI (2 mM).
  10. Place both pipettes in the micromanipulator pipette holders.
  11. Apply gentle positive pressure, place the pipettes in the bath, focus on their tips, and move them down, one by one, toward the surface of the slice.
  12. Stop moving the pipettes before approaching the cell; ensure that the cell body is below the whole-cell pipette.
  13. Set the Multiclamp-700B Commander Channels 1 and 2 to voltage-clamp mode and correct the pipette offset.
  14. Apply a seal test, a -10 mV voltage step, to both pipettes.
  15. Approach the cell body with the first pipette by moving it along the z-axis until a dimple appears on the membrane.
  16. Release the positive pressure and apply gentle suction while monitoring the current response to the voltage step until the gigaohmic seal forms.
  17. Move the holding voltage to -70 mV and correct fast capacitance using automatic correction of the Commander.
  18. Apply gentle and brief suction pulse with a syringe to break into whole-cell configuration (or by mouth). The successful break-in is evident by an abrupt increase in the amplitude of capacitative transients in response to -10 mV voltage steps.
  19. Switch the Commander to the Current Clamp mode.
  20. Correct the Bridge.
  21. A Current Clamp whole-cell recording is established for pipette 1.
- **Measuring action currents along the axon**
  1. After establishing the Current Clamp whole-cell recording with pipette 1, wait 15 min to allow SBFI diffusion to the cell.
  2. Focus the NeuroCCD-SMQ camera on the axon. L5 cell axons are the only fine processes emerging from the soma opposite the apical dendrite and exhibiting distinctive Na^+^ transients (Fleidervish et al., 2010; Baranauskas et al., 2013). Some neurons were examined live using a two-photon microscope after the physiological experiment. In these cells, axons were easily discerned from basal dendrites due to their lack of spines, confirming earlier observations during recording.
  3. Acquire an 8 ms long single frame image with a NeuroCCD-SMQ camera using the NeuroPlex software. Use 385 nm high-intensity LED device as a light source and collect emission using the U-MNU2 filter set. The 8 ms long exposure time is sufficient to obtain a high-quality image while minimizing light intensity that might induce photo-dynamic damage to the cell.
  4. Find a point of interest along the axon. The first point of interest could be at ~10 µm from the edge of the soma if the backpropagation velocity has to be measured; in experiments designed to measure the forward propagation, the first point of interest might be more distal than the presumed trigger zone, ~30–40 µm from the edge of the soma.
  5. Bring pipette 2 to the point of interest (Figure 1b).
  6. Touch the axon with the pipette 2. Ensure the touch by taking one or more single frames with the NeuroCCD-SMQ camera. The proper touch could be established by fine-tuning the pipette 2 position, with no suction applied to the pipette.
  7. Deliver 2–7 ms long current steps via pipette 1. Increase the current step amplitude until it will be sufficient to elicit an AP.
  8. Deliver 600 steps of 1.5× threshold amplitude at 2–5 Hz frequency. Record the voltage with pipette 1 and current with pipette 2.
  9. Store the recording file for further analysis.
  10. Find another point of interest (Figure 1c). It should be 10–20 µm further distally from the previous point of interest to ensure a significant time delay difference.
  11. Repeat steps 5–9 for 6–10 points of interest within 10–150 µm of the axonal length.
    **Figure 1.**
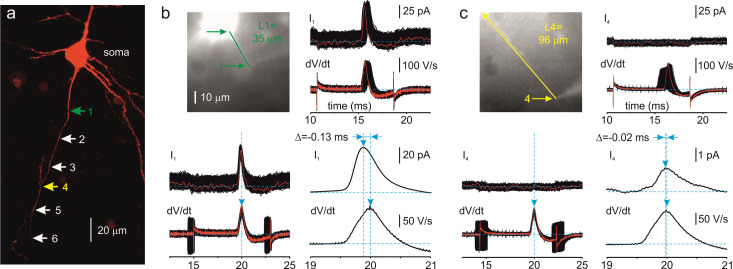
Measuring the distances and propagation delays between the cell body (soma) and distinct points along the axon. a. Maximal intensity Z-stack projection of 39 two-photon optical sections through part of a Layer 5 pyramidal neuron filled with SBFI (red) obtained at a wavelength of 760 nm. The arrows indicate sites along the axon where loose patch recordings were performed. The sites are color coded as follows: 1 (green): 35 μm, 4 (yellow): 96 µm from the edge of the soma. b. Measurement of the propagation delay for site 1. *Left*, the same neuron as seen during the imaging experiment with the NeuroCCD–SMQ camera. The green arrows indicate the region corresponding to the edge of the soma and the site from where action currents were obtained. *Right, top*, 600 somatic dV/dt sweeps and corresponding axon currents from site 1, unaligned and following alignment using the time of dV/dtmax as the reference point. *Right, bottom*, ensemble average of the somatic dV/dt (bottom trace) and axonal current (top trace). Note that the peak of the axonal action current precedes the peak of the somatic dV/dt by 130 µs. c. Measurement of the propagation delay for site 4. For details, see b. Note that, at the distance of 96 µm, the somatic dV/dt peak precedes the peak of the action current by 20 µs.
- **Neuronal morphology (optional)**
  1. Turn on the Ultima IV 2P microscope and perfuse aCSF through the slice mini chamber.
  2. Set the temperature under the objective to 32 °C using the temperature controller.
  3. Transfer the slice containing SBFI-labeled cell from the slice mini chamber of the Olympus microscope setup to the slice mini chamber of the Ultima 2P microscope and put a platinum grid to pin down the slice.
  4. Using a low-resolution Live Scan at 740 nm excitation wavelength, identify the SBFI-labeled neuron and place it in the center of the field of a 40× or 60× Olympus water-immersion lens.
  5. Create a Z series of 20–40 images at 0.5–1.0 μm depth intervals (Figure 1a).
  6. Open the resulting Z-stack in ImageJ to obtain morphometric data.

## Data analysis

1. Measuring the axonal length between the soma and pipette 2:
  - Open the fluorescent image containing the cell body and the proximal axon, taken using the NeuroCCD-SMQ camera, in ImageJ (Figure 1b, 1c).
  - Measure the total SBFI fluorescence along the soma-axon axis using the Analyze-Plot Profile command. The half-difference of the somatic and axonal fluorescence should be assigned as zero point for the axonal length measurements (Baranauskas et al., 2013).
  - Draw the straight line or lines connecting the zero point and the location of pipette 2 over the axon.
  - Use the Pythagorean theorem to find the distance between the two points.
2. Measuring the time latency between pipettes 1 and 2:
  - Open the data file containing 600 consecutive sweeps of somatic voltage and currents collected from an axonal location in Clampfit 10 (Figure 1b, c).
  - Go to the Analysis Window Toolbar and push the *Arithmetic* button. In the *Arithmetic* window dialogue, select the Active Window, Somatic Vm signal, and All visible traces. Differentiate the somatic voltage sweeps using the expression T{VISIBLE} = diff(T{VISIBLE}), to obtain the dV/dt sweeps.
  - Align all sweeps and signals using the dV/dt positive peaks as a reference point using the Analyze-Time Shift-Align peaks command (replace wrapped samples with zeros). The alignment of sweeps will compensate for unavoidable AP jitter.
*Note: Current is proportional to differentiated voltage. Thus, we can measure the time lag between the peak dV/dt and action current using cursors 1 and 2.*
  - Average the dV/dt and action current sweeps and measure the time delay between their peaks.
3. Repeat steps 1–2 for other files collected.
4. Plot the distance of the pipette 2 from the soma as a function of time delay (Figure 2).
  **Figure 2.**
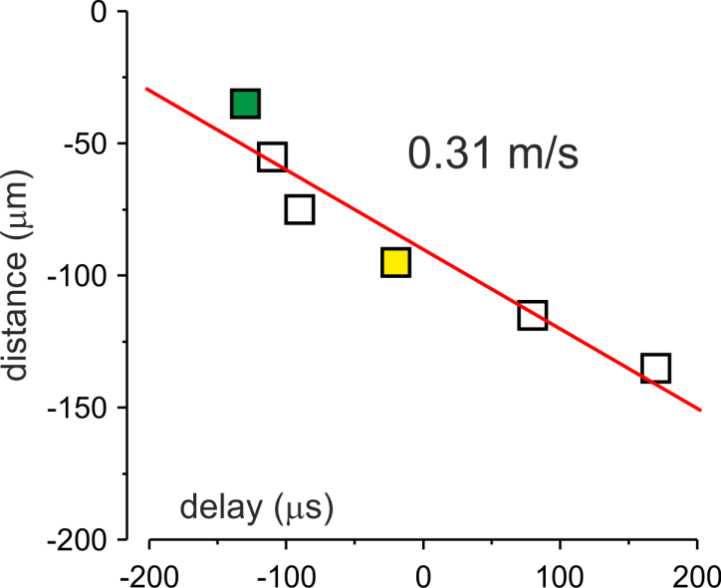
Measuring the propagation velocity. Distance from the edge of the soma plotted against the delay of action current generation. Each dot corresponds to the mean delay to the onset of the somatic action potential (AP) at a given location in the same neuron shown in Figure 1. The red line is a linear fit of the data. Notice that the AP initiates in a region at ~35 μm from the soma and propagates forward with a velocity of 0.31 m/s.
5. Determine the pipette 2 location at which the time delay is maximal. Designate this location as the AP initiation zone. Note that the spatial precision of this measurement depends on the relative distances between the locations at which the action current has been measured.
6. All data points from sites proximal to the AP initiation zone correspond to time delays of the backpropagating AP. All points distal to the AP initiation zone are latencies of the forward propagating AP.
7. The slope of the linear fit of these datasets gives a velocity of back- and forward-propagating AP.
